# Factors and outcomes of pediatric sepsis under an antimicrobial stewardship program in a tertiary care teaching hospital in Thailand: a retrospective cohort study

**DOI:** 10.1017/ash.2026.10811

**Published:** 2026-08-04

**Authors:** Theerapong Seesin, Suvaporn Anugulruengkitt, Chankit Puttilerpong

**Affiliations:** 1 Department of Pharmacy Practice, Chulalongkorn University Faculty of Pharmaceutical Sciences, Bangkok, Thailand; 2 Department of Pediatrics, Chulalongkorn University Faculty of Medicine, Bangkok, Thailand; 3 Center of Excellence for Pediatric Infectious Diseases and Vaccines, Chulalongkorn University, Bangkok, Thailand; 4 Department of Pharmacy Practice, Center of Excellence in Bioactive Resources for Innovative Clinical Applications, Chulalongkorn University Faculty of Pharmaceutical Sciences, Bangkok, Thailand

## Abstract

**Objective:**

To investigate the factors associated with mortality and the outcomes of an antimicrobial stewardship program (ASP) in pediatric patients with sepsis.

**Design:**

Retrospective cohort study.

**Setting:**

A tertiary care teaching hospital in Thailand.

**Patients:**

Pediatric patients with sepsis.

**Methods:**

This study reviewed electronic medical records of pediatric patients. A computer-generated random sequence was used to select 240 patients who met the inclusion criteria for the analysis.

**Results:**

Between January 1, 2021, and December 31, 2024, a total of 1,777 patients were enrolled in the ASP. Of 240 eligible patients, males accounted for 56.7% of the cohort, with a median age of 1.0 year (interquartile range 0–6). Positive blood cultures were identified in 72 patients (30.0%). ASP interventions identified antibiotic-related problems in 93 patients (38.8%), with primary physicians adhering to ASP recommendations in 55 cases (59.1%). Septic shock was the only factor significantly associated with 30-day mortality (adjusted odds ratio 4.064; 95% confident interval: CI 1.265–13.061; *P* = .019). Mortality rates were not different between groups regarding ASP compliance or blood culture results. The day of therapy/1,000 PD for vancomycin and meropenem were significantly lower in the compliance group compared to the non-compliance group (81.9 vs 135.4, *P* = .026; and 169.9 vs 260.3, *P* = .012, respectively).

**Conclusions:**

Septic shock was the only factor associated with 30-day mortality. ASP help detected antibiotic-related problems and reduced meropenem and vancomycin consumption in pediatric patients with sepsis.

## Introduction

Antibiotics use often leads to various complications including adverse reactions and antibiotics resistance.^1^ A 2019 study utilizing predictive models estimated that 4.95 million deaths globally were associated with antibiotic resistance across 204 countries.^2^ Antimicrobial stewardship programs (ASPs) are developing strategies to address these issues since 2006.^1^ Afterward, there were several studies regarding ASPs implementation in adults whereas studies in children remains limited. A systematic review included 113 pediatric ASP studies conducted in 2020 found the interventions employed in the studies varied, with prospective audit and feedback being the most common. Most of the studies were conducted in the United States, where ASPs have been well implemented. However, there is a notable gap in studies from low-and middle-income countries. The outcome measures assessed in the studies included antibiotic consumption, changes in inappropriate antibiotic prescribing, compliance among prescribing physicians, and shifts in antimicrobial resistance. However, many of these studies lacked clinical outcome measures and failed to examine the factors associated with these outcomes.^3^ A study examining factors associated with mortality in the pediatric intensive care unit (PICU) identified several key factors, including a fluid overload, the mechanical ventilation support, the vasoactive drugs use, and the presence of congenital anomalies. However, the study did not include ASPs as a factor in its analysis.^4^

Sepsis is a life-threatening condition associated with high mortality rates in children. One study found an in-hospital mortality rate of 7.1% in higher-resource settings and 28.5% in lower-resource settings. Despite the high mortality rates, research on the implementation of ASPs in pediatric sepsis remains limited.^5^

Unfortunately, blood cultures for bacteria often yield negative results in patients with sepsis, complicating the selection of appropriate antibiotics and potentially affecting clinical outcomes. A study conducted in a PICU examined the association between culture results and patient outcomes found a high proportion of culture-negative sepsis (86.6%), but mortality was higher in patients with positive cultures compared to those with negative cultures (43% vs 20%). However, this study did not focus on the impact of ASPs.^6^

Thailand is a low-middle income country where antibiotics resistance is increasing. A study reported 87,751 infections in Thailand caused by resistant bacteria in 1,023 hospitals, leading to an extended hospital stay of 3.24 million days, 38,481 deaths in one year.^7^ The ASPs implementation in pediatrics in Thailand remains limited. A study in pediatric units in a medical school hospital in Thailand assessing the impact of a prospective audit and feedback ASP was conducted as a descriptive retrospective analysis. The study found a significant reduction in day of therapy (DOT) for vancomycin and colistin during the postASP implementation. However, there was no observed effect on length of stay (LOS) or mortality between the pre and postASP periods. Importantly, this study did not examine factors and effects of ASP compliance on clinical outcomes, particularly in those with sepsis, and did not focus on culture results.^8^

The objectives of this study were to investigate the factors associated with mortality and the outcomes of an ASP in pediatrics with sepsis in a tertiary care teaching hospital in Thailand, with particular emphasis on antibiotics consumption and LOS regarding compliance with the ASP and blood culture results.

## Methods

### Study design and setting

This study was a retrospective cohort analysis of pediatric inpatient data obtained from a tertiary care teaching hospital in central Thailand. The hospital has 200 pediatric inpatient beds and receives referrals from provincial hospitals nationwide. The pediatric ASP at this institution has implemented a prospective audit and feedback model since 2017. The ASP team comprises infectious disease (ID) pediatricians and ID pharmacists.

Each morning, ID pharmacists generate a list of patients receiving selected antibiotics commonly used to treat resistant organisms, including piperacillin–tazobactam, meropenem, vancomycin, colistin, sulbactam, amikacin, and fosfomycin, for review during ASP rounds. The ASP team conducts ward rounds twice weekly, on Mondays and Thursdays, focused on assessing the appropriateness of antibiotic therapy. When antibiotic-related problems are identified, the ASP team provides recommendations to the attending physicians. Physician compliance with these interventions is monitored within 24 hours.

### Data collection

Data were extracted from admission records stored in the hospital’s computerized database. Pediatric patients aged 0–17 years who met the diagnostic criteria for sepsis, defined as the presence of a condition fulfilling the systemic inflammatory response syndrome (SIRS) criteria, were included. Eligible patients were those admitted to pediatric units and who had received one or more of the selected antibiotics for at least 48 hours.

Exclusion criteria included patients with incomplete clinical data, those who developed allergic reactions leading to discontinuation of selected antibiotics, patients who refused medical treatment, patients who died within 48 hours after receiving selected antibiotics, and patients under long-term palliative care.

### Sample size calculation

The reported mortality rate of pediatric sepsis ranges from 4% to 50%.^9^ According to standard recommendations for logistic regression analysis, a minimum of 10 outcome events is required per predictor variable.^10^ Based on this guideline and given that 12 factors were selected from the literature and clinical relevance namely age, sex, nutritional status, Pediatric Comorbidity Index (PCI) score, septic shock, mechanical ventilation use, vasoactive agent use, postoperative status, fluid overload, quick Pediatric Logistic Organ Dysfunction-2 (qPELOD-2) score, blood culture result, and ASP compliance, the minimum sample size was estimated accordingly.

$$n=(EPV^{*}\kappa )/\pi$$

When EPV = event per variables, *κ* = covariates, and *π* = event rate.

$$n=(10^{\;*}12)/0.5=240$$

### Sampling and data extraction

A computer-generated sequence was used to random all hospital numbers of pediatric patients enrolled in the ASP between January 1, 2021, and December 31, 2024. Patient records were reviewed until 240 cases that met the inclusion and exclusion criteria were obtained.

Patient age was defined as the age (in years) on the first day of hospital admission. Sex was categorized as male or female. Nutritional status was classified as well-nourished, undernourished, or overweight based on the World Health Organization charts.

The PCI score, comprising 24 specific conditions with a total possible range of 0–48 points, was used to provide a more accurate assessment of mortality risk compared with comorbidity presence alone. The qPELOD-2 score, which includes three clinical parameters; tachycardia, hypotension, and altered mentation (Glasgow Coma Scale (GCS) < 11) was used to evaluate organ dysfunction on the first day of admission.

Fluid overload was identified based on physician documentation or diuretic therapy. Blood culture results were classified as positive or negative according to the first blood sampling. ASP compliance was defined as full acceptance of all recommendations made by the ASP team.

Other variables were recorded as binary (yes/no), including septic shock (physician-diagnosed), use of mechanical ventilation of any type or duration, administration of vasoactive agents (eg, norepinephrine, epinephrine, or dopamine), and postoperative status prior to sepsis onset.

### Data analysis

Data were analyzed using IBM SPSS Statistics version 30.0 (IBM Corp., Armonk, NY, USA). Categorical variables were analyzed using the χ^2^ test or Fisher’s exact test, as appropriate. The Kolmogorov–Smirnov test was used to assess the normality of continuous variables. Since most data were non-normally distributed, medians and interquartile ranges (IQRs) were reported, and the Mann–Whitney U test was used for group comparisons.

Variables with a *P* value < .25 in univariate analysis were considered candidates for inclusion in multivariable analysis. Binary logistic regression was performed to calculate crude odds ratios (ORs) and adjusted odds ratios (aORs) to identify factors associated with 30-day mortality.

Antibiotic consumption was measured using days of therapy (DOT) standardized to DOT per 1,000 patient-days (DOT/1,000 PD), which accounts for dosing variability in pediatric populations. The Mann–Whitney *U* test was used to compare DOT/1,000 PD and length of stay between ASP compliance and non-compliance groups, and between culture-positive and culture-negative sepsis groups.

## Results

### Patient characteristics

**Figure 1.**
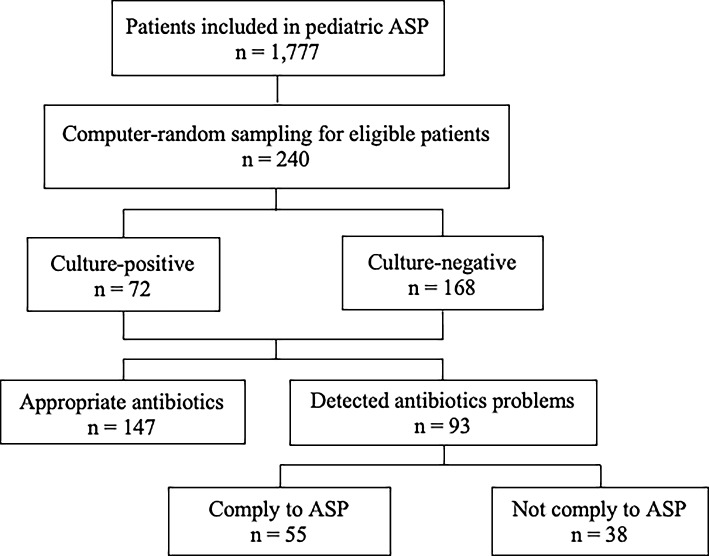
Flow diagram of pediatric patients included in the study.

Between January 1, 2021, and December 31, 2024, a total of 1,777 pediatric patients were enrolled in the ASP. As many patients had multiple admissions, 6,223 tracking lists were generated for ASP rounds during the period. Using a computer-generated random sequence, 240 pediatric sepsis cases that met the inclusion criteria were selected for analysis (Figure 1).

Among these patients, 136 (56.7%) were male and 104 (43.3%) were female. The median age was 1 year. The majority had a good nutritional status (67.5%). Nearly all patients (99.6%) had at least one comorbidity, with a median PCI score of 5.0.

Postoperative status at any time prior to diagnosis of sepsis was observed in 134 patients (55.8%). Mechanical ventilation was required in 158 patients (65.8%). Other indicators of sepsis severity were less frequent, including septic shock (27.5%), vasoactive agent use (27.5%), and fluid overload (41.3%). The median GCS was 15.0, and the median qPELOD-2 score was 1.0. Most baseline characteristics were not statistically different between the ASP compliance and non-compliance groups, or between the culture-positive and culture-negative groups (Table 1).

**Table 1.** Patients’ characteristics Table 1 long description.

Characteristics	Total n = 240	Culture results (n = 240)			Total n = 93	ASP interventions (n = 93)		
		Positive n = 72	Negative n = 168	*P*-value		Compliance n = 55	Non-compliance n = 38	*P*-value
Sex (%)				.609^a^				.240^a^
Male	136 (56.7)	39 (54.2)	97 (57.7)		58 (62.4)	37 (67.3)	21 (55.3)	
Female	104 (43.3)	33 (45.8)	71 (42.3)		35 (37.6)	18 (32.7)	17 (44.7)	
Age, years, median (IQR)	1.0 (0.0–6.0)	1.0 (0.0–3.0)	1.0 (0.0–8.0)	.275^c^	1.0 (0.0–9.0)	1.0 (0.0–7.0)	2.5 (0.0–9.25)	.224^c^
Age category (%)				.684^a^				.014^b^
Neonate	32 (13.3)	11 (15.3)	21 (12.5)		12 (12.9)	5 (9.1)	7 (18.4)	
Infant	95 (39.6)	30 (41.7)	65 (38.7)		38 (40.9)	29 (52.7)	9 (23.7)	
Child	113 (47.1)	31 (43.1)	82 (48.8)		43 (46.2)	21 (38.2)	22 (57.9)	
Nutritional status (%)				.590^b^				.692^b^
Undernourished	71 (29.6)	19 (26.4)	52 (31.0)		30 (32.3)	17 (30.9)	13 (34.2)	
Well-nourished	162 (67.5)	50 (69.4)	112 (66.7)		61 (65.5)	36 (65.5)	25 (65.8)	
Overweight	7 (2.9)	3 (4.2)	4 (2.4)		2 (2.2)	2 (3.6)	0 (0)	
Presence of comorbidity (%)	239 (99.6)	72 (100.0)	167 (99.4)	1.000^b^	92 (98.9)	54 (98.2)	38 (100.0)	1.000^b^
PCI score, median (IQR)	5.0 (3.0–7.0)	6.0 (3.0–7.0)	4.0 (3.0–6.8)	.041^c^	5.0 (3.0–7.0)	5.0 (3.0–7.0)	5.0 (4.0–8.0)	.205^c^
Postoperative status (%)	134 (55.8)	39 (54.2)	95 (56.5)	.734^a^	57 (61.3)	32 (58.2)	25 (65.8)	.459^a^
Fluid overload (%)	99 (41.3)	31 (43.1)	68 (40.5)	.710^a^	38 (40.9)	25 (45.5)	13 (34.2)	.278^a^
GCS, median (IQR)	15.0 (10.0–15.0)	15.0 (10.0–15.0)	11.5 (10.0–15.0)	.185^c^	11.0 (10.0–15.0)	11.0 (10.0–15.0)	12.5 (10.0–15.0)	.649
qPELOD-2 score, median (IQR)	1.0 (0.0–1.0)	0.0 (0.0–1.0)	1.0 (0.0–1.0)	.099^c^	1.0 (0.0–1.0)	1.0 (0.0–1.0)	0.0 (0.0–1.0)	.311
Ventilator use (%)	158 (65.8)	38 (52.8)	120 (71.4)	.005^a^	63 (67.7)	35 (63.6)	28 (73.7)	.308^a^
Septic shock (%)	66 (27.5)	24 (33.3)	42 (25.0)	.185^a^	21 (22.6)	11 (20.0)	10 (26.3)	.474^a^
Vasoactive agent use (%)	66 (27.5)	22 (30.6)	44 (26.2)	.488^a^	22 (23.7)	11 (20.0)	11 (28.9)	.318^a^
Source of infections (%)				<.001^b^				.945^a^
Known sources	167 (69.6)	62 (86.1)	105 (62.5)		64 (68.8)	38 (69.1)	26 (68.4)	
Unknown source	73 (30.4)	10 (13.9)	63 (37.5)		29 (31.2)	17 (30.9)	12 (31.6)	

a χ^2^.

b Fisher’s exact test.

c Mann–Whitney *U* test.

Of the 240 patients included in the study, 72 (30.0%) had positive blood culture results. Among isolates, 73.6% were classified as non-resistant, while 26.4% were identified as resistant organisms, including methicillin-resistant *Staphylococcus aureus* and *Staphylococcus epidermidis*, carbapenem-resistant *Klebsiella pneumoniae*, carbapenem-resistant *Pseudomonas aeruginosa*, carbapenem-resistant and pan-drug resistant *Acinetobacter baumannii*, and extended-spectrum β-lactamase–producing *Escherichia coli*.

Antibiotic-related problems requiring ASP intervention were identified in 93 cases (38.8%). These included the need for antibiotic discontinuation (25.0%), narrowing of the antibiotic spectrum (9.2%), dosage or administration modification (2.9%), and adjustment of treatment duration (1.7%). Among these cases, attending physicians adhered to ASP recommendations in 55 patients (59.1%). Reasons for non-adherence included lack of clinical improvement, which required the continued use of the existing broad-spectrum antibiotics (13.2%) and physician preference (13.2%); in 73.6% of cases, no specific reason for non-compliance was documented.

### Clinical outcomes and antibiotic utilization

The 30-day mortality rate was 6.7%. There was no statistically significant difference in 30-day mortality or length of hospital stay between the ASP compliance and non-compliance groups. However, patients in the culture-negative group had a significantly shorter hospital stay compared with those in the culture-positive group (*P* = .002), while 30-day mortality did not differ between the two groups.

Across all participants, the total duration of hospitalization amounted to 15,703 patient-days. Meropenem represented the most frequently used antibiotic, with a utilization rate of 204.8 DOT/1,000 PD. The DOT/1,000 PD for both meropenem and vancomycin was significantly lower in the ASP compliance group than in the non-compliance group (*P* = .012 and *P* = .026, respectively). In contrast, piperacillin/tazobactam use was significantly higher among patients with culture-negative sepsis (*P* = .008) (Table 2).

**Table 2.** Clinical outcomes and antibiotic utilization Table 2 long description.

Outcomes	Total n = 240	Culture results (n = 240)			Total n = 93	ASP interventions (n = 93)		
		Culture-positive n = 72	Culture-negative n = 168	*P*-value		Compliance n = 55	Non-compliance n = 38	*P*-value
30-day mortality (%)	16 (6.7)	3 (4.2)	13 (7.7)	.405^a^	4 (4.3)	3 (5.5)	1 (2.6)	.642^a^
Length of stay, median (IQR)	38.5 (23.0–74.8)	51.0 (27.3–120.5)	36.5 (21.8–66.3)	.002^b^	45.0 (26.0–85.0)	41.0 (22.0–78.0)	48.0 (28.8–92.0)	.284^b^
Patient-day (PD)	15,703	7,023	8,680		6,413	3,820	2,593	
DOT/1,000 PD								
Piperacillin-tazobactam	116.8	92.0	137.1	.008^b^	106.7	101.0	113.0	.341^b^
Meropenem	204.8	172.1	231.2	.685^b^	206.5	169.9	260.3	.012^b^
Vancomycin	124.0	114.6	131.6	.254^b^	103.5	81.9	135.4	.026^b^
Colistin	67.1	60.2	72.6	.199^b^	70.0	64.9	77.5	.895^b^
Sulbactam	45.8	46.8	44.9	.319^b^	43.8	40.1	49.4	.545^b^
Amikacin	46.6	38.6	53.1	.542^b^	57.9	51.6	67.1	.412^b^
Fosfomycin	4.1	0.0	7.4	.255^b^	5.3	8.9	0.0	1.000^b^
Other antibiotics	194.5	153.6	227.5	.900^b^	220.6	242.1	189.0	.441^b^
Total selected antibiotics	609.1	524.1	677.9	.569^b^	593.6	518.3	704.6	.766^b^
All antibiotics	803.6	677.8	905.4	.344^b^	814.3	760.5	893.6	.138^b^

a Fisher’s exact test.

b Median DOT/1,000 PD were compared by using Mann–Whitney *U* test.

### Factors associated with 30-day mortality

Univariate analysis was performed to compare characteristics between survivors and non-survivors. Variables with a *P* value < .25 were considered for inclusion in the multivariable logistic regression model. These variables included age, qPELOD-2 score, fluid overload, septic shock, and vasoactive agent use.

Prior to multivariable analysis, multicollinearity testing was conducted. High variance inflation factor (VIF) values were observed for septic shock (VIF = 3.728) and vasoactive agent use (VIF = 3.758), suggesting potential collinearity between these variables. To address this issue, only septic shock was retained for inclusion in the final multivariable logistic regression model.

The multivariable logistic regression analysis identified septic shock as the only factor independently associated with 30-day mortality, with an aOR of 4.064 (95% CI: 1.265–13.061) (Table 3).

**Table 3.** Factors associated with 30-day mortality Table 3 long description.

Factors	30-day deceased n = 16	30-day survived n = 224	*P*-value	Crude odds ratio (95% CI)	*P*-value	Adjusted odds ratio (95% CI)	*P*-value
Age, median (IQR)	0.0 (0–2.75)	1.0 (0.0–6.0)	.156^a^	1.101 (0.951–1.273)	.197^d^	1.124 (0.957–1.321)	.154^d^
qPELOD-2 score, median (IQR)	1.0 (0.0–1.0)	0.0 (1.0–1.0)	.223^a^	0.722 (.385–1.352)	.308^d^	1.095 (.519–2.311)	.812^d^
Fluid overload (%)	10 (62.5)	124 (55.4)	.074^b^	2.528 (0.887–7.202)	.083^d^	1.796 (0.599–5.384)	.296^d^
Septic shock (%)	9 (56.3)	57 (25.4)	.016^c^	3.767 (1.341–10.578)	.012^d^	4.064 (1.265–13.061)	.019^d^

a Mann–Whitney *U* test.

b χ^2^.

c Fisher’s exact test.

d Binary logistic regression.

## Discussion

The 30-day mortality rate of 6.7% observed in this study was within the range of 4%–50% reported in previous studies on pediatric sepsis.^9^ This relatively low mortality rate may reflect the effectiveness of sepsis management and the implementation of the ASP at our institution. As a super-tertiary teaching hospital in Thailand, our findings align with prior evidence reporting a hospital mortality rate of 7.1% in higher-resource settings.^5^

No significant difference in 30-day mortality was observed between culture-positive and culture-negative sepsis (4.2% vs 7.7%, *P* = .405). This contrasts with previous research, which found higher mortality in culture-positive sepsis compared with culture-negative sepsis among PICU patients (43% vs 20%, *P* = .004).^6^ The difference may be attributed to the predominance of non-resistant organisms (73.6%) in our cohort, which are typically more responsive to empiric therapy. Moreover, empiric antibiotic regimens for culture-negative sepsis in our setting appeared to be appropriate.

Similarly, no difference in mortality was detected between ASP compliance and non-compliance groups. This finding may be influenced by the limited number of patients requiring ASP intervention, which could have reduced the statistical power to detect a difference.

Septic shock recognized as a severe manifestation of sepsis that leads to multiorgan dysfunction and high mortality was the only factor independently associated with 30-day mortality in our analysis (aOR 4.064; 95% CI: 1.265–13.061; *P* = .019). Management aspects of septic shock, including fluid resuscitation, vasopressor use, and corticosteroid therapy, extend beyond the ASP’s primary focus on antimicrobial optimization and were not within the scope of this study. Because vasoactive agent use was strongly correlated with septic shock, it was excluded from the multivariable model due to multicollinearity. Nevertheless, it should be considered an important clinical factor for future ASP improvement, especially in efforts to integrate broader sepsis management strategies beyond antibiotic use. Our findings are consistent with previous studies demonstrating associations between septic shock, vasoactive agent use, and increased mortality in pediatric sepsis.^4^

Other factors such as age, qPELOD-2 score, and fluid overload were not significantly associated with mortality in our study. The qPELOD-2 score was used in place of the full PELOD-2 score due to data availability. While the PELOD-2 score, which includes 10 parameters across five organ systems, is a well-validated predictor of mortality in critically ill children, the simplified qPELOD-2 score has also demonstrated predictive value for mortality in children with suspected infection.^11^ However, no significant association was observed between qPELOD-2 score and 30-day mortality in our cohort.

A previous study reported that fluid overload was significantly associated with both the duration of mechanical ventilation and mortality in pediatric patients with sepsis.^12^ In that study, patients were categorized into two groups based on fluid status: ≤10% and >10% fluid overload, calculated from cumulative fluid intake and output within the first 48 hours of admission. In contrast, due to data limitations, fluid overload in our study was identified based on diagnostic records and the administration of diuretics, which may have affected the results. In our cohort, fluid overload was not significantly associated with mortality.

An association between congenital anomalies and increased mortality in pediatric sepsis has been reported in a previous study.^4^ In the present study, comorbidity was assessed using the PCI score, which encompasses 24 conditions including congenital anomalies and has been validated as an effective risk-adjustment tool in pediatric epidemiologic research.^13^ However, no significant association between the PCI score and mortality was observed in our cohort.

Our ASP detected antibiotic-related problems in 93 patients (38.8%), while 147 patients (61.2%) received appropriate antibiotic therapy. The 59.1% compliance rate of attending physicians is consistent with other study following prospective audit and feedback pediatrics ASP implementation which achieved acceptance rate about 59%.^14^ These results highlight ongoing challenges for ASPs and underscore the need for strategies that enhance physician engagement, such as feedback sessions and case-based learning.

The LOS was significantly longer in culture-positive sepsis compared with those with culture-negative cases (median LOS: 51.0 vs 36.5 d, *P* = .002), likely reflecting greater illness severity and presence of resistant organisms requiring prolonged antibiotic therapy. Correspondingly, the DOT/1,000 PD of piperacillin–tazobactam was higher in culture-negative sepsis which may indicate empirical therapy in the setting where nosocomial infections commonly require coverage for *Staphylococci* spp. and *Pseudomonas aeruginosa.* In contrast, DOT/1,000 PD was lower among culture-positive patients, suggesting the impact of ASP intervention to stop piperacillin-tazobactam use and shift toward documented therapy (DOT/1,000 PD: 92.0 vs 137.1, *P* = .008).

ASP compliance was associated with significant reductions in antibiotic consumption, with meropenem use decreasing from 260.3 to 169.9 DOT/1,000 PD (*P* = .012) and vancomycin use decreasing from 135.4 to 81.9 DOT/1,000 PD (*P* = .026) when compared to non-compliance group. These results are consistent with previous studies demonstrating the effectiveness of ASPs in reducing antibiotic use.^3^ A prior study in the same institution also reported decreases in vancomycin use (from 58.5 to 40.2 DOT/1,000 PD; *P* = .038), colistin (from 36.3 to 13.8 DOT/1,000 PD; *P* = .026), and meropenem (from 126.8 to 111.2 DOT/1,000 PD; *P* = .467) after ASP implementation without compliance assessment.^8^

The findings on factors associated with mortality and antibiotic-related problems may contribute to the development of innovative ASP aimed at improving outcomes. There were limitations including confounding by indication bias, and immortal time bias leading to limited event per variable. Future research should focus on conducting cost-outcome analyses that incorporate clinical and humanistic outcomes. Additionally, the effectiveness of strategies to control antibiotic resistance remains a critical area for further investigation.

## Conclusion

Septic shock was the factor independently associated with 30-day mortality in pediatric sepsis under the ASP. Culture results and ASP compliance did not affect mortality; however, culture-positive sepsis was associated with prolonged hospitalization and reduced piperacillin–tazobactam consumption. The ASP effectively identified antibiotic-related problems, and physician compliance with ASP led to significant reductions in meropenem and vancomycin use.

## Data Availability

The findings data are not publicly available due to privacy or ethical restrictions. The data are available from the corresponding author upon reasonable request.

## Table 1. Long description

The table compares patient characteristics and ASP interventions. It has 240 total patients divided into culture results (positive and negative) and ASP interventions (compliance and non-compliance). The table includes characteristics such as sex, age, nutritional status, presence of comorbidity, PCI score, postoperative status, fluid overload, GCS, qPELOD-2 score, ventilator use, septic shock, vasoactive agent use, and source of infections. Row 1: Sex (percent), Male 136 (56.7), Positive 39 (54.2), Negative 97 (57.7), P-value .609, Total 93, Compliance 55, Non-compliance 38, P-value .240. Row 2: Sex (percent), Female 104 (43.3), Positive 33 (45.8), Negative 71 (42.3), Total 35 (37.6), Compliance 18 (32.7), Non-compliance 17 (44.7). Row 3: Age, years, median (IQR), Total 1.0 (0.0–6.0), Positive 1.0 (0.0–3.0), Negative 1.0 (0.0–8.0), P-value .275, Total 1.0 (0.0–9.0), Compliance 1.0 (0.0–7.0), Non-compliance 2.5 (0.0–9.25), P-value .224. Row 4: Age category (percent), Neonate 32 (13.3), Positive 11 (15.3), Negative 21 (12.5), P-value .684, Total 12 (12.9), Compliance 5 (9.1), Non-compliance 7 (18.4), P-value .014. Row 5: Age category (percent), Infant 95 (39.6), Positive 30 (41.7), Negative 65 (38.7), Total 38 (40.9), Compliance 29 (52.7), Non-compliance 9 (23.7). Row 6: Age category (percent), Child 113 (47.1), Positive 31 (43.1), Negative 82 (48.8), Total 43 (46.2), Compliance 21 (38.2), Non-compliance 22 (57.9). Row 7: Nutritional status (percent), Undernourished 71 (29.6), Positive 19 (26.4), Negative 52 (31.0), P-value .590, Total 30 (32.3), Compliance 17 (30.9), Non-compliance 13 (34.2), P-value .692. Row 8: Nutritional status (percent), Well-nourished 162 (67.5), Positive 50 (69.4), Negative 112 (66.7), Total 61 (65.5), Compliance 36 (65.5), Non-compliance 25 (65.8). Row 9: Nutritional status (percent), Overweight 7 (2.9), Positive 3 (4.2), Negative 4 (2.4), Total 2 (2.2), Compliance 2 (3.6), Non-compliance 0 (0). Row 10: Presence of comorbidity (percent), Total 239 (99.6), Positive 72 (100.0), Negative 167 (99.4), P-value 1.000, Total 92 (98.9), Compliance 54 (98.2), Non-compliance 38 (100.0), P-value 1.000. Row 11: PCI score, median (IQR), Total 5.0 (3.0–7.0), Positive 6.0 (3.0–7.0), Negative 4.0 (3.0–6.8), P-value .041, Total 5.0 (3.0–7.0), Compliance 5.0 (3.0–7.0), Non-compliance 5.0 (4.0–8.0), P-value .205. Row 12: Postoperative status (percent), Total 134 (55.8), Positive 39 (54.2), Negative 95 (56.5), P-value .734, Total 57 (61.3), Compliance 32 (58.2), Non-compliance 25 (65.8), P-value .459. Row 13: Fluid overload (percent), Total 99 (41.3), Positive 31 (43.1), Negative 68 (40.5), P-value .710, Total 38 (40.9), Compliance 25 (45.5), Non-compliance 13 (34.2), P-value .278. Row 14: GCS, median (IQR), Total 15.0 (10.0–15.0), Positive 15.0 (10.0–15.0), Negative 11.5 (10.0–15.0), P-value .185, Total 11.0 (10.0–15.0), Compliance 11.0 (10.0–15.0), Non-compliance 12.5 (10.0–15.0), P-value .649. Row 15: qPELOD-2 score, median (IQR), Total 1.0 (0.0–1.0), Positive 0.0 (0.0–1.0), Negative 1.0 (0.0–1.0), P-value .099, Total 1.0 (0.0–1.0), Compliance 1.0 (0.0–1.0), Non-compliance 0.0 (0.0–1.0), P-value .311. Row 16: Ventilator use (percent), Total 158 (65.8), Positive 38 (52.8), Negative 120 (71.4), P-value .005, Total 63 (67.7), Compliance 35 (63.6), Non-compliance 28 (73.7), P-value .308. Row 17: Septic shock (percent), Total 66 (27.5), Positive 24 (33.3), Negative 42 (25.0), P-value .185, Total 21 (22.6), Compliance 11 (20.0), Non-compliance 10 (26.3), P-value .474. Row 18: Vasoactive agent use (percent), Total 66 (27.5), Positive 22 (30.6), Negative 44 (26.2), P-value .488, Total 22 (23.7), Compliance 11 (20.0), Non-compliance 11 (28.9), P-value .318. Row 19: Source of infections (percent), Known sources 167 (69.6), Positive 62 (86.1), Negative 105 (62.5), P-value <.001, Total 64 (68.8), Compliance 38 (69.1), Non-compliance 26 (68.4), P-value .945. Row 20: Source of infections (percent), Unknown source 73 (30.4), Positive 10 (13.9), Negative 63 (37.5), Total 29 (31.2), Compliance 17 (30.9), Non-compliance 12 (31.6).

Navigate back to Table 1..

## Table 2. Long description

The table presents clinical outcomes and antibiotic utilization data for 240 participants, divided into culture-positive and culture-negative groups, and further into ASP compliance and non-compliance groups. The table has 12 rows and 11 columns. Column headers are Outcomes, Total n = 240, Culture results (n = 240), Culture-positive n = 72, Culture-negative n = 168, P-value, ASP interventions (n = 93), Total n = 93, Compliance n = 55, Non-compliance n = 38, and P-value. Row labels include 30-day mortality (percent), Length of stay (median IQR), Patient-day (PD), DOT/1,000 PD, and various antibiotics. Row 1: 30-day mortality (percent), 16 (6.7), 3 (4.2), 13 (7.7), 0.405, 4 (4.3), 3 (5.5), 1 (2.6), 0.642. Row 2: Length of stay (median IQR), 38.5 (23.0-74.8), 51.0 (27.3-120.5), 36.5 (21.8-66.3), 0.002, 45.0 (26.0-85.0), 41.0 (22.0-78.0), 48.0 (28.8-92.0), 0.284. Row 3: Patient-day (PD), 15,703, 7,023, 8,680,, 6,413, 3,820, 2,593, . Row 4: DOT/1,000 PD,, , , , , , , , . Row 5: Piperacillin-tazobactam, 116.8, 92.0, 137.1, 0.008, 106.7, 101.0, 113.0, 0.341. Row 6: Meropenem, 204.8, 172.1, 231.2, 0.685, 206.5, 169.9, 260.3, 0.012. Row 7: Vancomycin, 124.0, 114.6, 131.6, 0.254, 103.5, 81.9, 135.4, 0.026. Row 8: Colistin, 67.1, 60.2, 72.6, 0.199, 70.0, 64.9, 77.5, 0.895. Row 9: Sulbactam, 45.8, 46.8, 44.9, 0.319, 43.8, 40.1, 49.4, 0.545. Row 10: Amikacin, 46.6, 38.6, 53.1, 0.542, 57.9, 51.6, 67.1, 0.412. Row 11: Fosfomycin, 4.1, 0.0, 7.4, 0.255, 5.3, 8.9, 0.0, 1.000. Row 12: Other antibiotics, 194.5, 153.6, 227.5, 0.900, 220.6, 242.1, 189.0, 0.441. Row 13: Total selected antibiotics, 609.1, 524.1, 677.9, 0.569, 593.6, 518.3, 704.6, 0.766. Row 14: All antibiotics, 803.6, 677.8, 905.4, 0.344, 814.3, 760.5, 893.6, 0.138.

Navigate back to Table 2..

## Table 3. Long description

The table presents data on factors associated with 30-day mortality, comparing deceased and survived patients. It includes columns for 30-day deceased (n=16), 30-day survived (n=224), P-value, Crude odds ratio (95% CI), P-value, Adjusted odds ratio (95% CI), and P-value. The factors analyzed are Age (median, IQR), qPELOD-2 score (median, IQR), Fluid overload (percent), and Septic shock (percent). Each row provides specific values for these factors. Notable trends include higher percentages of fluid overload and septic shock in deceased patients compared to survivors. The adjusted odds ratio for septic shock is significantly high, indicating a strong association with 30-day mortality.

Navigate back to Table 3..
